# Chikungunya Virus Infection in Traveler to Australia

**DOI:** 10.3201/eid1303.060761

**Published:** 2007-03

**Authors:** Julian D. Druce, Douglas F. Johnson, Thomas Tran, Michael J. Richards, Christopher J. Birch

**Affiliations:** * Victorian Infectious Diseases Reference Laboratory, North Melbourne, Victoria, Australia; † Royal Melbourne Hospital, Parkville, Melbourne, Victoria, Australia

**Keywords:** Alphavirus, PCR, Chikungunya virus, diagnosis, detection, Australia, letter

**To the Editor:** Chikungunya is a mosquitoborne alphavirus in the family *Togaviridae*. Recently, a chikungunya virus epidemic that affected thousands of persons occurred in islands in the southwestern Indian Ocean, including Mauritius and Reunion (*1*). An outbreak is ongoing in India, and cases are being exported to many other countries (*2*–*4*) The likelihood of importation of exotic infectious agents into Australia increased during events such as the March 2006 Commonwealth Games in Melbourne. Urgent diagnosis of rarely seen infections is a travel health challenge, particularly when serologic tests used for diagnosis in areas with high prevalence are not locally available. Only 1 previously diagnosed case of Chikungunya virus infection has been reported in Australia; it involved importation of the virus from Indonesia to Darwin in 1989 (*5*). We report a second case of infection with this virus.

A 59-year-old man came to a hospital emergency department in North Melbourne, Australia, on March 12, 2006, 5 days after traveling from Mauritius for the Commonwealth Games. He reported a 2-day history of acute swelling and erythema of the left leg and associated malaise. Twenty-four hours earlier, severe lumbar back pain and arthralgias that involved the lower limbs had developed, with associated fevers, rigors, and headache.

The patient had a temperature of 39°C, bilateral conjunctivitis, tender and markedly swollen Achilles tendons, a swollen left ankle, and a maculopapular rash that involved the left forefoot and anterior portion of the shin. Initial blood examinations showed leukopenia (leukocyte count 1.8 × 10^9^/L, lymphocyte count 0.2 × 10^9^/L), mild thrombocytopenia (platelet count 10^5^ × 10^9^/L), and abnormal liver function test results (alanine aminotransferase 133 IU/mL, γ-glutamyl transpeptidase 141 IU/mL, bilirubin 7 µmol/L). Malaria blood films and dengue serologic results were negative. A validated, in-house, generic alphavirus reverse transcription–PCR (RT-PCR) showed positive results 24 hours after collection of blood when the patient was admitted.

The patient received supportive treatment and was discharged from the hospital 3 days after admission, at which time leukopenia and thrombocytopenia had improved. The patient had fully recovered on review 1 week after discharge.

For virus isolation, plasma and leukocyte fractions were placed onto Vero E6 cells and incubated at 37°C for 5 days. Cells were observed daily for virus-specific cytopathic effects. A virus isolate was obtained after 4 days of cell culture. A 10-µL volume of supernatant from infected cells was applied to a carbon-coated grid, stained with phototungstic acid, and examined by electron microscopy. This procedure showed virus with morphology similar to Togavirus (data not shown).

Chikungunya virus was identified by a heminested RT-PCR for the nonstructural protein 4 gene. First-round primers were AlphaF1 (GenBank accession no. NC004162, nt 6942–6961, 5′-CSATGATGAARTCHGGHATG-3′) and AlphaR (nt 7121–7141, 5′-CTATTTAGGACCRCCGTASAG-3′). Second-round primers were AlphaF2 (nt 7480–7501) (5′-TGGNTBAAYATGGAGGTIAAG-3′) and AlphaR. Sequencing of the second-round product identified the virus. A 339-bp fragment (GenBank accession no. DQ678928) had 97% identity with the African prototype strain S27 isolated in Tanzania (Tanganyika) in 1953 (AF369024) and 100% identity with viral sequences from Reunion Island in 2006 (DQ443544).

Knowledge of distant epidemics aids clinical recognition of infections not commonly seen in Australia. Websites and electronic bulletins (e.g., Promed) are a conduit of information. On the basis of this case-patient, those in Australia became more aware of the chikungunya virus epidemics affecting the islands of the southwestern Indian Ocean.

Laboratory diagnosis of chikungunya virus infection is usually serologic. However, alphavirus infections not endemic to Australia are unlikely to be diagnosed serologically because specific assays are generally available only for those viruses known to circulate in Australia. Because viremia of alphaviruses is brief, success of RT-PCR depends on early admission and clinical recognition of infection (*6*).

Rapid establishment of a definitive diagnosis had substantial benefits that included management of the febrile patient, reduced need for further investigations, and better prognosis. Infection caused by an introduced arbovirus may have important public health implications in Australia. Because immunity in the Australian population is unlikely, consideration must be given to the potential for transmission of the virus to caregivers and the local community. Chikungunya virus is commonly spread by mosquitoes of the genera *Aedes*, including *Aedes aegypti*, *Ae*. *furcifer-taylori*, *Ae*. *luteocephalus*, *Ae*. *albopictus*, and *Ae*. *dalzieli* (*7*). The Australian Ross River and Barmah Forest alphaviruses are spread by many species of *Aedes* and *Culex* (*8*). Because some local species could transmit chikungunya virus, necessary steps should be taken to ensure containment when a patient is viremic.

This case highlights the potential for exotic viruses to be introduced into Australia by visitors or returning travelers and the utility of molecular testing for their rapid detection. The generic nature of the RT-PCR enabled detection of an alphavirus with subsequent specific identification by sequencing. Rapid identification and differentiation in a public health setting minimized the potential for spread of the virus.
